# Statistical Gems and Cautions From the Statistical Advisory Board of Clinical Oral Implants Research

**DOI:** 10.1111/clr.70033

**Published:** 2025-09-04

**Authors:** Cláudio Mendes Pannuti, Hélio Doyle Pereira da Silva, Naichuan Su, Kar Yan Li, Lisa J. A. Heitz‐Mayfield

**Affiliations:** ^1^ School of Dentistry University of São Paulo São Paulo Brazil; ^2^ Dental Research Division, Department of Periodontology Guarulhos University Guarulhos Brazil; ^3^ Department of Oral Public Health, Academic Centre for Dentistry Amsterdam (ACTA) University of Amsterdam and Vrije Universiteit Amsterdam Amsterdam the Netherlands; ^4^ Clinical Research Centre, Faculty of Dentistry the University of Hong Kong Hong Kong China; ^5^ International Research Collaborative, Oral Health and Equity, School of Human Anatomy and Biology The University of Western Australia Crawley Western Australia Australia; ^6^ School of Dentistry, Faculty of Medicine and Health The University of Sydney Sydney New South Wales Australia

**Keywords:** bias, controlled clinical trial, data analysis, dental implants, sample size, statistics

## Abstract

**Objective:**

Statistics is fundamental in implant dentistry research, playing a crucial role in study design, data analysis, and interpretation of findings. This editorial, authored by members of the Statistical Advisory Board of Clinical Oral Implants Research, aims to highlight common statistical pitfalls encountered in submitted manuscripts and provide practical recommendations to enhance the quality of statistical reporting.

**Materials and Methods:**

In this editorial, study design considerations, sample size calculation, appropriate selection of statistical tests, multiplicity issues, handling hierarchical data structures, and best practices for data presentation are discussed. The importance of pre‐registering research protocols, adhering to pre‐specified analyses, and reporting effect sizes alongside *p*‐values is emphasised. Additionally, common misinterpretations of statistical significance and the risks of extrapolating findings beyond their intended scope are addressed*.*

**Results:**

Statistical errors remain prevalent in the scientific literature, affecting the validity of results and evidence‐based decision making.

**Conclusions:**

By following guidelines, authors can improve the robustness and reproducibility of their research, ultimately contributing to higher‐quality evidence in implant dentistry. Guidance for researchers, reviewers, and readers in handling statistical challenges is presented aiming to ensure that studies published in Clinical Oral Implants Research meet the highest methodological standards.

## 1 Introduction

Research in implant dentistry and statistical analyses has become sophisticated, making the involvement of a statistician in the research team a prerequisite for quality research today. Understanding the complexities of statistical analyses can be challenging for both readers and clinicians. Hence, this editorial has been prepared to assist both the esteemed authors and readers of *Clinical Oral Implants Research* (COIR) by outlining the key points to consider and look for in the study design, analysis, and reporting of research findings. The best clinical decisions are based on evidence, and insights from statistical advisors are essential in this process.

Statistics are fundamental to the entire research process, from study design to the final reporting of results. Despite their importance, manuscripts submitted to scientific journals frequently contain methodological and statistical errors. Common issues include underpowered studies, inappropriate selection of statistical tests, misinterpretation of statistical significance, and inadequate data presentation, among others (Diong et al. 2018; Thiese et al. 2015, 2017; Arreola et al. 2020; Chambrone et al. 2023).

These errors often stem from a fundamental misunderstanding and misapplication of statistical principles. Additionally, the widespread availability of statistical software, often used by inexperienced researchers, has exacerbated this problem. Such errors can lead to misleading conclusions, significantly impacting the quality of papers in biomedical journals and, ultimately, evidence‐based decision making and patient safety.

Many high‐impact journals, such as *Clinical Oral Implants Research*, include statistical advisors as an integral part of the review process with the aim of assisting authors to improve the quality of their publications. Indeed, evidence suggests that statistical advisors improve the quality of papers in medical journals (Gardner and Bond 1990; Murray 1988). However, the frequency of errors remains high. Statistical advisors frequently encounter mistakes in study design, data analysis, reporting, and interpretation of results.

Therefore, this editorial aims to highlight the most common mistakes encountered in study design, data analysis, reporting and interpretation, and to provide guidance to authors of *Clinical Oral Implants Research* (COIR) when planning a study and preparing a manuscript for submission. While it is not possible to address all issues, we hope that the recommendations will assist authors and enhance the quality of studies published in COIR, ultimately leading to better clinical practices and improved patient outcomes.

## 2 Study Design

### 2.1 Always Consult a Statistician Before the Study Starts

Involving a statistician before starting a study can significantly improve the quality of the research protocol, making it more robust and ensuring that the study design and data analysis plan meet ethical standards. Statisticians can assist researchers in formulating clear and testable hypotheses, ensuring that the research questions are precise and the study objectives are feasible. Without early statistical involvement, incorrect decisions in study design, randomization, matching methods, and covariate selection may occur. Additionally, consulting a statistician for sample size calculation is crucial to avoid an underpowered study.

### 2.2 Use Protocol Guidelines or Reporting Guidelines Early in the Study Planning

Studies should be conducted according to a *research protocol*, a document that outlines the study's rationale, hypothesis, aims, methods, and ethical considerations (Chan et al. 2025). Consulting a guideline when drafting the protocol helps ensure that no critical methodological elements are omitted—such as how exposures and outcomes will be measured, or how the sample size was calculated. Using such guidelines early helps researchers anticipate key design requirements and ensure that all essential elements are clearly defined in the protocol. This, in turn, enhances the transparency and reproducibility of the study.

When available, researchers should follow formal *protocol development guidelines*. For randomized clinical trials, the SPIRIT 2025 Statement (Standard Protocol Items: Recommendations for Interventional Trials) provides a comprehensive checklist covering eligibility criteria, randomization procedures, outcome definitions, statistical methods, and ethical considerations, among other items (Chan et al. 2025). For systematic reviews, the PRISMA‐P Statement outlines key elements that should be addressed in review protocols, including rationale, eligibility criteria, data sources, and synthesis methods (Moher et al. 2015).

For study designs that lack a protocol‐specific guideline—such as observational studies or case series—*reporting guidelines* like STROBE (Strengthening the Reporting of Observational Studies in Epidemiology) (von Elm et al. 2007) or CARE (Consensus‐based Clinical Case Reporting) (Gagnier et al. 2014) can serve as practical surrogates. These checklists, while intended for reporting, highlight essential elements that should be planned in advance and included in the protocol.

Using protocol or reporting guidelines early in the research process—not just at the manuscript writing stage—can help prevent many of the methodological and statistical issues frequently encountered by the statistical advisors of COIR. We strongly encourage authors to incorporate these tools from the earliest stages of study planning.

### 2.3 Specify the Statistical Analysis Plan, Including Superiority or Non‐Inferiority Hypothesis

The *statistical analysis plan* (SAP) is a critical component of the research protocol and should be clearly defined prior to data collection. The SAP should specify the statistical methods to be used for each type of outcome (e.g., categorical, continuous, time‐to‐event), the analysis population (e.g., intention‐to‐treat or per‐protocol), and whether any covariate adjustment is planned—particularly for key prognostic variables identified a priori (read Section 3.2 for more information). The SAP must also describe how missing data will be handled, any planned strategies for controlling multiplicity (Section 3.3), the statistical significance threshold (e.g., two‐sided alpha of 0.05), the confidence intervals to be reported, and the statistical software to be used (Gamble et al. 2017). A well‐developed SAP promotes transparency, minimizes the risk of data‐driven analyses and selective reporting (Sections 2.5 and 5.3), and ensures alignment with the pre‐specified study objectives and registered protocol.

Importantly, the SAP should also specify whether the trial is designed to demonstrate superiority or non‐inferiority. In *superiority trials*, the objective is to demonstrate that the new intervention is significantly better than the comparator. The null hypothesis assumes no difference between the treatments. A statistically significant result (typically *p* < 0.05) allows rejection of this hypothesis in favor of the alternative, indicating that a difference exists between the treatments. For example, the multicenter trial by Payer et al. (2020), published in *Clinical Oral Implants Research*, tested a clear superiority hypothesis regarding the effect of systemic antibiotics on patient‐reported outcomes and post‐surgical complications in implant therapy with guided bone regeneration. Accordingly, the sample size was calculated based on a superiority trial. However, no statistically significant difference was observed between groups, indicating that antibiotics provided no additional benefit.

In contrast to superiority trials, *non‐inferiority trials* aim to demonstrate that a new treatment is not meaningfully worse than the standard of care, within a pre‐defined margin of acceptable difference, called the non‐inferiority margin (Δ) (Cavalcante et al. 2025; Food and Drug Administration 2016). A non‐inferiority hypothesis is indicated when the new intervention offers other relevant benefits—such as reduced cost, less morbidity/side effects, or easier administration—but may not necessarily be more effective. In such trials, if the data show that the difference between treatments is smaller than Δ, the null hypothesis can be rejected and non‐inferiority can be concluded (Cavalcante et al. 2025; Food and Drug Administration 2016). Unlike superiority trials, where the effect size is primarily used for sample size calculation, non‐inferiority trials rely heavily on the non‐inferiority margin for both design and interpretation. Therefore, defining Δ appropriately is critical and should be clinically justifiable and clearly stated in the protocol. If the margin is too wide, there is a risk of accepting a treatment that is actually worse. If it is too narrow, the required sample size may be unfeasibly large (Cavalcante et al. 2025; Piaggio et al. 2012). A good example of a non‐inferiority trial is the study by Hämmerle et al. (2023), which compared a collagen matrix to autogenous connective tissue grafts for soft tissue augmentation. Although the collagen matrix was not expected to provide greater crestal mucosal thickness gain, it offers other advantages such as reduced morbidity and surgical time. The trial used a non‐inferiority margin of 0.5 mm for crestal mucosal thickness gain but yielded inconclusive results, as non‐inferiority could not be demonstrated.

To understand how to structure a complete statistical analysis plan (SAP), readers are encouraged to consult the Guidelines for the Content of Statistical Analysis Plans by Gamble et al. (2017). The guideline includes a comprehensive checklist of items to be addressed in a clinical trial SAP, along with detailed explanations and practical examples provided in the E‐Appendix 2 of their publication.

### 2.4 Provide Detailed Information About Sample Size Calculation

Both the protocol and the manuscript should contain a section describing how the sample size was calculated. This section should provide details such as the type I error rate (alpha), statistical power (1 − beta, where beta is the type II error rate), and the effect size (Schulz and Grimes 2005a, 2005b; Tu and Gilthorpe 2012). The *effect size* (ES) is commonly defined as the *observed* magnitude of the difference between groups, calculated after data collection (Durlak 2009). However, in the context of sample size calculation—which occurs before the study begins—effect size refers to the smallest *expected* difference between groups that would be considered meaningful. It should be based on the study's primary outcome and guided by a pilot study or a previous study that used the same outcome (Cook et al. 2018). For continuous outcomes, the ES is typically based on the difference between group means and is often expressed as a standardized effect size, such as Cohen's d. For studies involving more than two groups or predictors, measures such as Cohen's f may be appropriate. For categorical outcomes, the ES is based on the difference between proportions and may be expressed as the risk ratio or odds ratio. The choice of ES metric must correspond to the planned statistical test and should be clearly specified (Durlak 2009).

Additionally, it is important to describe whether the test is one‐ or two‐tailed, and if factors such as losses to follow‐up, non‐response, and design effects were considered. Importantly, the effect size should be established a priori—that is, before the study begins. Incomplete descriptions of sample size calculation that only include alpha and power are misleading.

When planning sample size for studies that intend to use regression models with covariate adjustment, researchers should account for the inclusion of key prognostic factors in the analysis. To minimize overfitting, at least 10–20 observations per covariate are generally recommended.

In some manuscripts where “statistically significant” results are not found, authors frequently include a *post hoc* power calculation. Sometimes, this is a request of the reviewer (Althouse 2021). This request aims to distinguish between true negatives and false negatives (where an actual effect exists, but the study failed to detect it due to insufficient sample size). However, *post hoc* power calculations are not recommended because they do not provide meaningful additional insights beyond what is already available from the *p*‐values. Instead, the correct approach should be a priori sample size calculation during the study design phase.

A good example of a well‐described sample size calculation can be found in a paper published in COIR, from Sanz‐Sánchez et al. (2024): “To calculate the minimum sample size needed, a power calculation was performed. The criterion for significance was set at 0.05 and the power at 0.80. Assuming a mean difference in bone level of 0.20 mm between groups, with a standard deviation (SD) of 0.157 (Canullo et al. 2010) and taking into consideration a foreseen 10% of likely drop‐outs; a total of 40 patients (20 per group) with a minimum of 40 implants were finally included.”

Readers interested in a deeper understanding of how to calculate sample size may refer to the book from Kieser (2020), who offers a detailed explanation of sample size calculation in different scenarios.

### 2.5 Register and Adhere to the Registered Protocol

Study protocols should be registered in a *public registry*. Adherence to the protocol is important because it ensures that the study follows a pre‐specified plan. This adherence minimizes the likelihood of data dredging or p‐hacking, where researchers might search through data for statistically significant results. Furthermore, alignment with the protocol prevents selective reporting. Discrepancies between the protocol and the manuscript constitute a form of reporting bias, such as selective outcome reporting (Chan et al. 2004; Souza et al. 2023), which is prevalent in implant studies (Sendyk et al. 2019). We recommend reviewers of COIR that they compare the protocol (if available) with the manuscript to identify discrepancies and prevent selective reporting.

To gain further insights into selective outcome reporting, we suggest consulting the Catalogue of Bias, from the Centre for Evidence‐Based Medicine (Catalogue Of Biases 2017), which offers a detailed explanation of this kind of bias.

## 3 Data Analysis

### 3.1 Ensure the Assumptions for Using a Parametric Test Are Met

*Parametric tests* are statistical tests that rely on certain underlying assumptions, such as: (a) the variable is normally distributed in the source population from which the sample is drawn and (b) the data sets are sampled from populations that have homogeneous variances (homocedasticity) (Riffenburgh and Gillen 2020; Motulsky 2017). When these assumptions are not met, *non‐parametric tests* should be used as an alternative to parametric tests.

There is ongoing discussion about the best methods for testing the assumption of normal distribution. Some authors recommend using statistical tests, such as the Shapiro–Wilk test, to verify whether data fit a normal distribution. Others suggest evaluating skewness and kurtosis, building histograms, or creating quantile‐quantile (Q–Q) plots to visually assess the distribution shape. The type of data is also an important consideration for certain authors. Additionally, some researchers advocate for using parametric tests in large studies even when the data are skewed, based on the central limit theorem, which states that the sampling distribution of the mean tends to approximate normality as sample size increases. (Altman and Bland 2009; Fagerland 2012; Kim and Park 2019; le Cessie et al. 2020; Vrbin 2022; Miot 2017).

Regardless of the approach taken to test these assumptions, it is crucial for authors to clearly explain their rationale for choosing between parametric and non‐parametric methods. It should be noted that some statistical tests are robust against slight violations of some assumptions. However, this discussion is beyond the scope of this editorial.

When the assumption of normality is not met, one alternative—before opting for non‐parametric methods—is to apply *data transformation* to better approximate a normal distribution. Common transformations include logarithmic, square root, or Box‐Cox transformations, depending on the characteristics of the data (Clark et al. 2016; West 2022). These methods can reduce skewness and make the data more suitable for parametric testing, provided they are used appropriately. However, log transformations are sensitive to zero values. They may also fail to adequately normalize the data if the extreme values—particularly in the distribution tails—remain skewed. This is often the case in the presence of outliers or biological heterogeneity that leads to skewed distributions (West 2022). When transformed data still do not approximate normality, non‐parametric methods may offer a more robust alternative. Therefore, any data transformation should be carefully justified, and the results should be interpreted with caution.

For a more in‐depth exploration of this topic, we recommend reading the book from Motulsky (2017), which provides a comprehensive review of the selection of statistical tests. Chapter 41 offers a thorough discussion on nonparametric tests and the choice between parametric and nonparametric tests.

### 3.2 Consider Adjustment for Baseline Covariates in Randomized Trials

In randomized controlled trials (RCTs), a common misconception is that randomization ensures balance of all baseline characteristics between treatment groups. While randomization does help prevent selection bias—that is, systematic differences in group allocation—it does not guarantee that all prognostic factors will be evenly distributed. In small samples, meaningful baseline imbalances can still arise by chance. Stratified randomization can help address this issue by balancing selected prognostic variables across groups, though only a limited number of variables can be used as strata. For example, stratification can be performed based on arch (maxilla vs. mandible) and history of periodontitis (yes vs. no). Even then, residual imbalances may remain, making it advisable to adjust for key baseline covariates in the analysis. Regression models that include these covariates can improve the precision of treatment effect estimates. However, in small trials, incorporating too many covariates increases the risk of overfitting. Therefore, adjustment should generally be limited to one or two important prognostic factors (Kernan et al. 1999), and this constraint should be considered during sample size planning.

Some researchers attempt to assess baseline balance by comparing *p*‐values for demographic and prognostic variables, typically presented in the baseline characteristics table. However, this practice is discouraged. A non‐significant *p*‐value (e.g., *p* > 0.05) does not imply equivalence between groups, but rather a lack of evidence to detect a difference—often due to low statistical power. Moreover, hypothesis testing for baseline characteristics contradicts the principle of randomization and may lead to misinterpretation (Altman and Doré 1990; de Boer et al. 2015).

Instead of testing for balance, researchers should consider adjusting for important prognostic factors by including them in a regression model (Holmberg and Andersen 2022). This is particularly important in small RCTs, where imbalances are more likely to occur (Hernández et al. 2004).

Guidance from regulatory agencies, including the U.S. Food and Drug Administration (FDA) and the European Medicines Agency (EMA), supports *prespecified covariate adjustment* to improve the precision of treatment effect estimates (Holmberg and Andersen 2022). Adjustments should be based on variables that are known or strongly suspected to be prognostic, measured prior to randomization, and defined in the protocol's statistical analysis plan. In these cases, adjusted regression models should be preferred over univariate comparisons to improve the robustness of the analysis.

### 3.3 Avoid Multiplicity in Data Analysis

*Multiplicity* issues arise when researchers conduct multiple comparisons, such as examining multiple outcomes, performing subgroup analyses, and conducting repeated interim analyses or repeated measurements of the same outcome (Schulz and Grimes 2005a, 2005b). These unplanned analyses can increase the overall type 1 error risk rate or, in other words, increase the likelihood of finding significant results purely by chance.

Authors should avoid unplanned subgroup analyses that were not pre‐specified in the protocol. Likewise, while trials typically have many outcomes, the protocol should have *only one pre‐specified primary outcome* (Hopewell et al. 2025). It is recommended to reduce the number of comparisons or apply multiplicity adjustments in data analysis to maintain the overall probability of false positives at a reasonable level. When the use of such adjustments is appropriate, they should be clearly specified in the study protocol and reported in the manuscript. The Bonferroni correction is widely used and offers strong control of the family‐wise error rate (i.e., the probability of making at least one type I error), but it is often criticized for being too conservative, increasing the risk of missing true effects (Hooper 2025). In contrast, the Benjamini–Hochberg procedure controls the false discovery rate (FDR)—the expected proportion of false positives among significant results (Benjamini and Hochberg 1995). It is less stringent and better suited for exploratory analyses or studies involving many comparisons. However, its tolerance for some false positives may be inappropriate in contexts where high certainty is essential, such as confirmatory studies or regulatory settings. The choice of whether to adjust, and which method to use, depends on the context, number of comparisons, and acceptable trade‐off between type I and type II errors (Hooper 2025; Li et al. 2017).

For more information on the multiplicity issue and on statistical methods to adjust for multiplicity, readers are encouraged to read Li et al. (2017).

### 3.4 Account for Hierarchical Data Structures

In dental implant research, data is often *hierarchical* (clustered or nested), that is, there is a hierarchical structure between the many levels: sites, implants, and patients (Fleming et al. 2013). In other words, sites are clustered within implants, and implants are clustered within patients. However, researchers often treat each site or implant as an independent observation, disregarding this clustering structure. This approach can lead to biased estimates of effect sizes, as the correlation among observations within clusters is not properly accounted for. This can result in overestimating or underestimating the true effect. Further, treating clustered data as independent observations can increase the risk of Type I error (Burnside et al. 2013).

When analyzing data from clustered structures, researchers must account for the hierarchical or nested nature of the data. This ensures that the analysis properly reflects the correlation among observations within clusters. Multilevel models (sometimes called hierarchical linear models) can be applied to analyze data with nested or hierarchical structures.

The study published in COIR by Manfredini et al. (2024) is a good example of how to analyze data with hierarchical structure. The authors used a three‐level linear mixed effects model for investigating the fixed effect of patient‐, prosthesis‐, and implant‐level variables on the marginal bone loss, therefore acknowledging the correlation between these different levels.

To gain a foundational understanding of multilevel modeling, we suggest reading Chapter 3 of “Multilevel Modelling for Public Health and Health Services Research” by Leyland and Groenewegen (2020). This chapter, “What is Multilevel Modelling?”, introduces core concepts and their applications in health research.

## 4 Reporting

### 4.1 Adhere to the Appropriate Reporting Guidelines

Detailed and transparent reporting is essential to ensure that study findings can be understood, critically appraised, and replicated. As noted in Section 2.2, one of the most effective ways to improve reporting quality is to follow *reporting guidelines* tailored to specific study designs. These guidelines provide checklists of essential elements that should be included, helping authors write their manuscripts with sufficient clarity and detail.

We recommend that authors follow the appropriate reporting guideline corresponding to their study type. For example, reporting of randomized controlled trials should follow CONSORT (Hopewell et al. 2025), observational studies should follow STROBE (von Elm et al. 2007), systematic reviews should follow PRISMA (Page et al. 2021), diagnostic accuracy studies should follow STARD (Bossuyt et al. 2015), and case reports may follow CARE (Gagnier et al. 2014). These and other guidelines are freely available through the EQUATOR Network (2024).

By adhering to these guidelines, authors improve not only the completeness and transparency of their reports, but also the likelihood that their manuscript will be understood, appropriately interpreted, and considered favorably during peer review. Reviewers of COIR are also encouraged to verify adherence to these guidelines during manuscript evaluation.

### 4.2 Use Appropriate Descriptive Statistics

For quantitative variables, provide measures of central tendency (mean or median) and measures of dispersion (standard deviation or interquartile range). For qualitative variables, report the full frequency distribution (number and percentage), rather than just the percentage.

It is recommended to use descriptive statistics that correspond to the statistical tests employed. When reporting the results of parametric tests, inform the mean and standard deviation (SD), formatted as “mean (SD)” rather than “mean ± SD.” Conversely, when employing non‐parametric tests, present the median and interquartile range. This approach ensures that the data presentation aligns with the chosen analytical methods.

Tables 1, 2, and 3 from the study by Kraus et al. (2022), published in COIR, appropriately present the mean (SD), median, first quartile (Q1) and third quartile (Q3), consistent with their use of the nonparametric Wilcoxon test to compare differences between groups for quantitative data.

For more insights into data presentation, the papers from Nieminen (2020) and Fah and Aziz (2006) offer comprehensive overviews on how to report descriptive statistics.

### 4.3 Report the Results Comprehensively

Traditionally, most health research has relied on hypothesis testing to examine relationships between variables, group differences, and the effectiveness of interventions, often emphasizing *p*‐values. However, clinicians and patients are not only interested in whether a treatment works but also in the *magnitude* of its effect. As discussed in Section 2.4, this magnitude is known as the *effect size* (ES) (Durlak 2009).

There is no direct correlation between statistical significance and the magnitude of an effect, which can lead to misinterpretations if only *p*‐values are reported. A *p*‐value of ≤ 0.05 does not necessarily indicate a clinically relevant effect, while a *p*‐value > 0.05 does not imply the absence of an important effect. This happens because *p*‐values can be affected by factors such as sample size and data variability.

Therefore, it is crucial to report ES measures (with their confidence intervals), alongside *p*‐values, to provide a more comprehensive understanding of the results (Tu and Gilthorpe 2012; Hopewell et al. 2025; Wasserstein et al. 2019). Commonly used ES metrics include Cohen's *d*, Glass's delta, and Cohen's *f* for continuous variables, and odds ratio and risk ratio for categorical variables. Providing effect sizes helps readers evaluate the practical significance of the findings. Further, reporting the 95% confidence interval is essential, as it indicates the precision of the estimated effect size and provides a range within which the true effect is likely to lie.

Researchers should also consider the scientific context by discussing their findings in relation to previous evidence, biological plausibility, and the associated costs, risks, and benefits. Additionally, the *observed* ES should be compared to the *expected* ES (read Section 2.4 on sample size calculation), which should be pre‐specified in the study protocol (Betensky 2019).

For supporting information on effect sizes, readers are encouraged to read the papers from Durlak (2009) and Glaros (2024).

### 4.4 Report *p*‐Values Correctly

Do not simply state that the results were “not statistically significant” or express the results as “NS” (Wasserstein et al. 2019). Similarly, avoid merely stating that an effect was “significant.” The American Statistical Association (ASA) encourages researchers to move away from using the term “statistical significance” (Wasserstein et al. 2019). Instead, when *p*‐values are utilized, they should be reported as continuous statistics (e.g., *p* = 0.06).

Furthermore, when reporting *p*‐values, present them with at least two figures, even for *p*‐values greater than 0.05 (e.g., *p* = 0.24 or *p* = 0.48, rather than *p* > 0.05). However, avoid excessive precision; *p*‐values less than 0.001 can be reported as *p* < 0.001 (Altman and Bland 1996).

### 4.5 Provide Clear and Informative Tables and Charts

Ensure that tables and charts are *self‐explanatory*, allowing readers to understand them without needing to read the rest of the article.

In general, it is better to organize tables with more rows than columns, as it is typically easier to read down than across. Use footnotes to explain abbreviations and symbols, to list confounding factors that have been adjusted for, and to provide details about the statistical tests and analyses used.

As commented on Section 4.2, for quantitative variables, provide measures of central tendency and measures of dispersion. For qualitative variables, report the frequency distribution (number and percentage). Further, as mentioned on Section 4.3, in addition to *p*‐values, include 95% confidence intervals and effect size estimates, such as odds ratios, risk ratios, or hazard ratios. Clearly indicate the statistical unit of analysis and specify the test used, along with its *p*‐value.

Bear in mind that other researchers may need your study's exact estimates for purposes like meta‐analyses or sample size calculation. Therefore, provide the exact numerical results even if you display the data in charts.

For building appropriate charts, consider using resources such as Harvard University's Data Visualization webpage (https://accessibility.huit.harvard.edu/data‐viz‐charts‐graphs#indocument), which provides tips and guidelines for constructing accessible graphs. Another helpful resource is Data to Viz (https://www.data‐to‐viz.com/), a tool that guides users in choosing suitable charts based on data type and comparison type. Additionally, we recommend Stephen Few's book “Show Me the Numbers” (Few 2012), which offers in‐depth guidance on designing effective tables and charts for clear and impactful communication.

## 5 Interpreting

### 5.1 Correctly Interpret *p*‐Values

As noted in item Section 4.3, it is incorrect to assume that a *p*‐value greater than 0.05 indicates the absence of an effect (e.g., that the treatment being studied is ineffective) (Tu and Gilthorpe 2012). Rather, it means that the result is inconclusive (Altman and Bland 1995). Similarly, when comparing two groups using a test of difference between means or proportions, a *p*‐value greater than 0.05 does not imply that the groups are “comparable,” “equivalent” or that they have “similar efficacy” (Hemming et al. 2022). This misinterpretation is especially problematic in smaller studies, which often lack sufficient statistical power to detect a clinically meaningful difference.

Therefore, it is crucial to explicitly acknowledge any uncertainty in the findings rather than misinterpreting non‐significant *p*‐values as evidence of no effect or similarity in effect between treatments.

### 5.2 Avoid Extrapolating Results

Researchers must exercise caution in interpreting findings, ensuring that conclusions are limited to only what is directly supported by the study data. Extrapolating results beyond the specific population or sample analyzed can compromise both the reliability and applicability of the findings. For instance, if a study investigates peri‐implantitis prevalence using a sample sourced from a dental school clinic, these results should not be generalized to the broader population, as a convenience sample is not representative of the entire community.

Similarly, results from randomized trials using surrogate outcomes—such as implant stability—should not be extrapolated to predict clinically relevant outcomes like implant failure unless validated by direct evidence (Pannuti et al. 2020). Making such assumptions risks overstating the study's implications and can lead to mistakes in clinical practice.

Finally, it is essential to differentiate correlation from causation (Stigler 2005). Although statistical associations can suggest relationships between variables, they do not confirm that one variable causes changes in another. Misinterpreting correlation as causation is a frequent error that may lead to unfounded conclusions and inappropriate applications in practice. Keeping conclusions grounded within the study's scope and data ensures conclusions are accurate and reliable.

### 5.3 Prevent Spin in the Interpretation of Results

In contrast to extrapolation (discussed in Section 5.2), which concerns extending findings beyond the scope of the study population or outcomes, *spin* refers to practices that distort the interpretation of results within the study itself. Spin can occur either intentionally or unintentionally and often presents study findings in a more favorable light than is supported by the data (Boutron et al. 2010). One type of spin, mentioned in Section 2.5, is *selective outcome reporting*, a form of reporting bias in which only favorable or statistically significant outcomes are disclosed (Chiu et al. 2017; Sendyk et al. 2019).

This practice undermines transparency and can mislead readers, reviewers, and policy‐makers. Boutron et al. (2010) classified spin strategies into three main categories:

- In the results section: spin may involve focusing on statistically significant secondary outcomes, within‐group comparisons, subgroup analyses, or analyses in modified populations (e.g., per‐protocol instead of intention‐to‐treat).
- In the discussion section: authors may interpret nonsignificant primary outcomes as evidence of treatment equivalence, overemphasize minor or secondary findings, or rule out adverse effects.
- In the conclusions section: may include claiming effectiveness without acknowledging the primary nonsignificant results, emphasizing alternative positive outcomes, or recommending clinical use despite inconclusive evidence.

For example, a dental implant trial may report no statistically significant difference between groups for the primary outcome (e.g., marginal bone loss), yet conclude that “the treatment is effective” based solely on within‐group improvements in the test group.

To prevent spin, authors should clearly acknowledge when primary outcomes are nonsignificant and avoid shifting emphasis to secondary or exploratory findings unless appropriately justified. Peer reviewers and editors are encouraged to check the consistency between the data and the manuscript's conclusions and to identify and address spin before publication.

## 6 Concluding Remarks

Transparency in reporting study hypothesis, methods, and results is essential for maintaining scientific rigor and integrity in dental implant research. A critical step in this process is ensuring that every aspect of the study, from sample size calculations to data analysis methods, is documented in detail and consistently aligned with the pre‐registered protocol. Adherence to the protocol minimizes the risk of selective reporting and *p*‐hacking, thereby preserving the credibility of the findings and enhancing reproducibility. Furthermore, providing comprehensive information when reporting methods and results enables peers to accurately evaluate and replicate the study. By adhering to these statistical and methodological practices, researchers will contribute to meaningful advances in the field and reinforce evidence‐based decision‐making in implant dentistry.

## Author Contributions

**Cláudio Mendes Pannuti:** conceptualization, writing – original draft, visualization, writing – review and editing, project administration. **Hélio Doyle Pereira da Silva:** writing – original draft, validation, writing – review and editing, visualization. **Naichuan Su:** writing – review and editing, visualization, validation. **Kar Yan Li:** validation, visualization, writing – review and editing. **Lisa J. A. Heitz‐Mayfield:** conceptualization, writing – review and editing, validation, supervision, visualization.

## Conflicts of Interest

The authors declare no conflicts of interest.

## Data Availability

Data sharing not applicable to this article as no datasets were generated or analyzed during the current study.
